# Fabrication of Eutectic Ga-In Nanowire Arrays Based on Plateau–Rayleigh Instability

**DOI:** 10.3390/molecules26154616

**Published:** 2021-07-30

**Authors:** Takashi Ikuno, Zen Somei

**Affiliations:** Tokyo University of Science, Katsushika, Tokyo 125-8585, Japan; mzeng@ikunolab.net

**Keywords:** nanowire, liquid metal

## Abstract

We have developed a simple method of fabricating liquid metal nanowire (NW) arrays of eutectic GaIn (EGaIn). When an EGaIn droplet anchored on a flat substrate is pulled perpendicular to the substrate surface at room temperature, an hourglass shaped EGaIn is formed. At the neck of the shape, based on the Plateau–Rayleigh instability, the EGaIn bridge with periodically varying thicknesses is formed. Finally, the bridge is broken down by additional pulling. Then, EGaIn NW is formed at the surface of the breakpoint. In addition, EGaIn NW arrays are found to be fabricated by pulling multiple EGaIn droplets on a substrate simultaneously. The average diameter of the obtained NW was approximately 0.6 μm and the length of the NW depended on the amount of droplet anchored on the substrate. The EGaIn NWs fabricated in this study may be used for three-dimensional wiring for integrated circuits, the tips of scanning probe microscopes, and field electron emission arrays.

## 1. Introduction

Gallium (Ga)-based liquid metals not only have excellent electrical and thermal conductivities with low viscosity and no toxicity [1,2], but are also environmentally friendly “green” materials because they can be recovered and recycled after use [3]. Among the Ga-based liquid metals, Ga-In eutectic alloy (EGaIn) is one of the most investigated liquid metals because it has a melting point lower than room temperature and is composed of binary elements. EGaIn is being applied to electrode materials for flexible, stretchable, and deformable devices [3,4,5,6,7,8,9,10,11,12,13,14,15] by taking advantage of the above intriguing properties. In parallel, fundamental research is being conducted on liquid metal properties and structure control from the viewpoints of electronics, electrochemistry, material sciences, additive manufacturing, and fluid mechanics [1,2].

When EGaIn is utilized for interconnections in various devices, the width of the interconnections is on the order of millimeters to sub-millimeters. In order to further expand the range of applications, it is necessary to improve the shape and position controllability. If EGaIn can be fabricated into quasi-one-dimensional anisotropic nanowires (NWs) with a sub-micron diameter, and if the NWs can be arranged in two-dimensional arrays on a flat substrate surface, they can be used for wiring in integrated circuits, tips for scanning probe microscopes [16,17,18], and field electron emission devices [19]. Previously, Ladd et al. fabricated EGaIn microwires with a diameter of approximately 200 μm by changing the distance between a syringe and a stage while injecting liquid metal into the stage from the syringe [20]. However, there is no report on the fabrication of two-dimensional arrays of EGaIn NWs.

In this study, we have developed a simple process to fabricate EGaIn NW arrays. This process is based on the Plateau–Rayleigh instability of viscoelastic materials. When a viscoelastic material is pulled by an external force, the thickness of the center of the viscoelastic material thins and the bridge is formed [21,22,23,24,25,26]. The bridge is unstable and breaks up into a series or array of small droplets in a “beads-on-thread” shape due to the Plateau–Rayleigh instability. When the bridge is broken down by additional pulling, we assumed that a submicron “thread,” namely an EGaIn NW, is formed at the breakpoint. Chiechi et al. previously reported that when a droplet of EGaIn was pulled to break up, the liquid metal changed from a hemispherical shape to a conical shape [27]. The lack of NW formation at the splitting point is thought to be due to the very slow pull. In order to utilize the Plateau–Rayleigh instability effectively, we thought it would be better to split the liquid metal at a fast rate [28,29].

## 2. Experimental

A polydimethylsiloxane (PDMS) film with a thickness of 2 mm was used as the substrate for the growth of EGaIn NWs. The PDMS film was obtained by the following procedure. First, the base and the agent (SYLGARD 184, The Dow Chemical Company, Midland, MI, USA) was mixed at a weight ratio of 10:1, and the mixture was poured on a glass plate with an enclosure at room temperature (RT). Second, the mixture on the glass plate was degassed by a rotary pump (RP). Third, the degassed mixture on the glass plate was heated at 150 °C for 10 min by a hot plate. The mixture was peeled away from the glass plate after cooling to RT.

**Figure 1:**
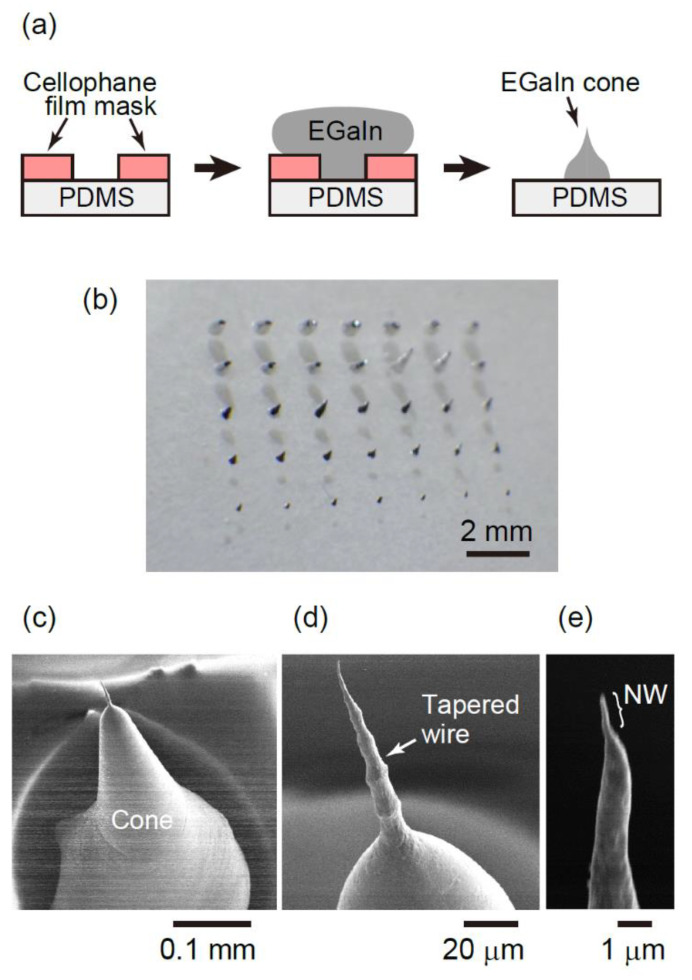
(**a**) Schematic illustration of NW fabrication process. (**b**) Photograph of EGaIn NW array (**c**–**e**) typical SEM images of the EGaIn corn and the protrusion at the corn.

After fabricating PDMS films, EGaIn NWs were fabricated by the thinning and breakup process shown in Figure 1a. First, 5 × 7 holes were fabricated at 1.5 mm intervals on a 20 mm square of a cellophane film using a laser processing machine (Podea-01, Podea Co., Ltd., Saitama, Japan). The thickness of the cellophane mask was 20 µm (# 110801, Toyo Corporation). The cellophane film with holes was put on the PDMS film with a 20 mm square. Second, EGaIn (300 µL), purchased from Kojundo Chemical Lab. Co., Ltd. (Saitama, Japan) (GAA02XB), was carefully dropped on the mask. Third, to attach the EGaIn with the PDMS film, the air in the space between the EGaIn droplet and the PDMS film was evacuated by a RP for 5 min. Finally, the mask was manually peeled off at RT with a tensile force of approximately 0.1 N and a peeling angle of approximately 45° at a speed of approximately four mm/s. Consequently, the array of EGaIn cone, upon each apex of which a NW was grown, was obtained.

The morphology and elemental composition of the obtained EGaIn cones and NWs were characterized by scanning electron microscopy (SEM) (S-4300, Hitachi-hitech, Tokyo, Japan) with energy dispersive X-ray spectroscopy (EDX).

## 3. Results and Discussion

Figure 1b shows a typical photograph of EGaIn cones fabricated on the PDMS film. Seven cones in the first, the second, the third, the fourth, and the fifth columns were obtained using the mask with the hole size of 0.57, 0.44, 0.36, 0.25, and 0.17 mm in diameter, respectively. We confirmed that EGaIn cones could be formed on the PDMS surface using the mask with the size of sub-millimeter diameter. Figure 1c–e provides typical SEM images of the cone fabricated using the 0.44 mm mask in diameter. The curvature of the cone was approximately 40 µm (Figure 1c). Surprisingly, there was a sharpened protrusion with a diameter of several µm at the apex of a cone. This protrusion was tapered and had a bamboo-like structure as shown in Figure 1d. This shape is similar to the “beads-on-thread” shape of viscoelastic materials [28]. A careful observation of the apex of the protrusion reveals a thin cylindrical structure with a diameter less than 1 µm at the tip of the bamboo-like microwire. We also confirmed that almost all cones shown in Figure 1b have similar wires at the apex. Thus, we found that sub-micron wires can be formed by the breakup of EGaIn droplets. As EGaIn is in the liquid phase at RT under atmospheric pressure, an EGaIn droplet is supposed to have a symmetrical conical or hemispherical shape [27] to minimize the surface energy. However, it interestingly exhibited a protrusion on the top. The fabrication mechanism will be discussed later.

**Figure 2:**
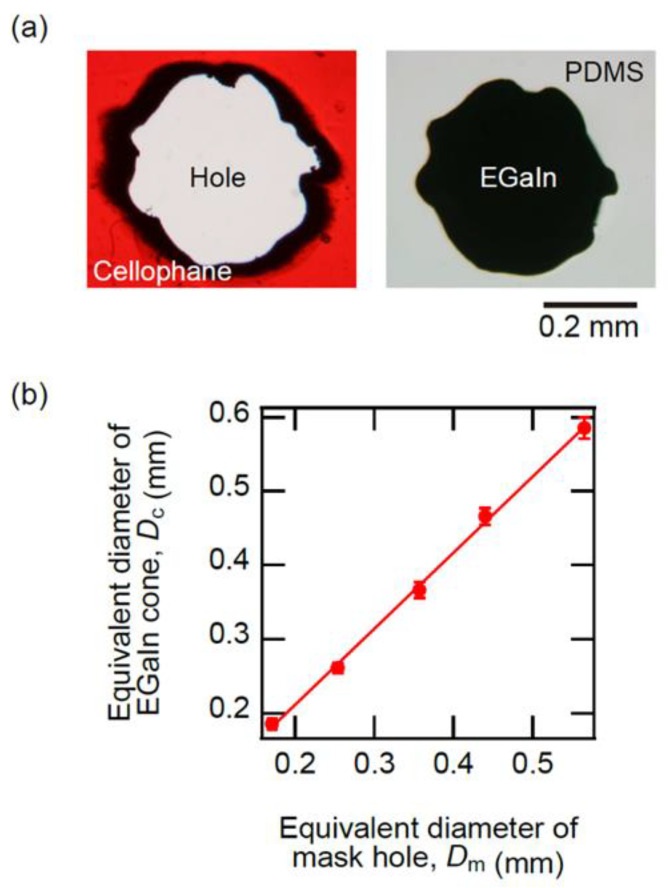
(**a**) Top-view photographs of the mask and the EGaIn corn fabricated by the mask. (**b**) The relationship between *D*_m_ and *D*_c_.

Next, we investigated the effect of the mask size on the footprint of EGaIn cones. The left image in Figure 2a shows a photograph of the mask holes with the size of 0.44 mm in diameter. The photograph was taken using a stereomicroscope with CCD (SZH, Olympus, Tokyo, Japan). The black edge of the hole is a region of burned cellophane film due to the laser irradiation when drilling holes. The open area was not a true circle because the laser processing machine does not have enough spatial resolution to engrave fine shapes of several tens of µm. The right image in Figure 2a shows a top-view photograph of the EGaIn cone fabricated using the mask indicated in the left image. Comparing the left image with the right image in Figure 2a, we can know the relationship between the area of the mask hole and the area of the cone bottom.

To clarify the relationship of the sizes, we used the equivalent diameter, *D*_m_, which is the diameter of the disk with the same area as the hole or the bottom. Figure 2b provides the relationship between the equivalent diameter of thecone bottom, *D*_c_, and the equivalent diameter of the mask hole, *D*_m_. We confirmed that *D*_c_ is proportional to *D*_m_, and the slope is almost 1. This means that the footprint of the EGaIn cone was almost equal to the area of the mask hole because the vacuum pump properly evacuated the air in the mask hole. Since the size of the EGaIn cone depends on the bottom area, the size of the EGaIn cone could be controlled by the size of the mask hole.

**Figure 3:**
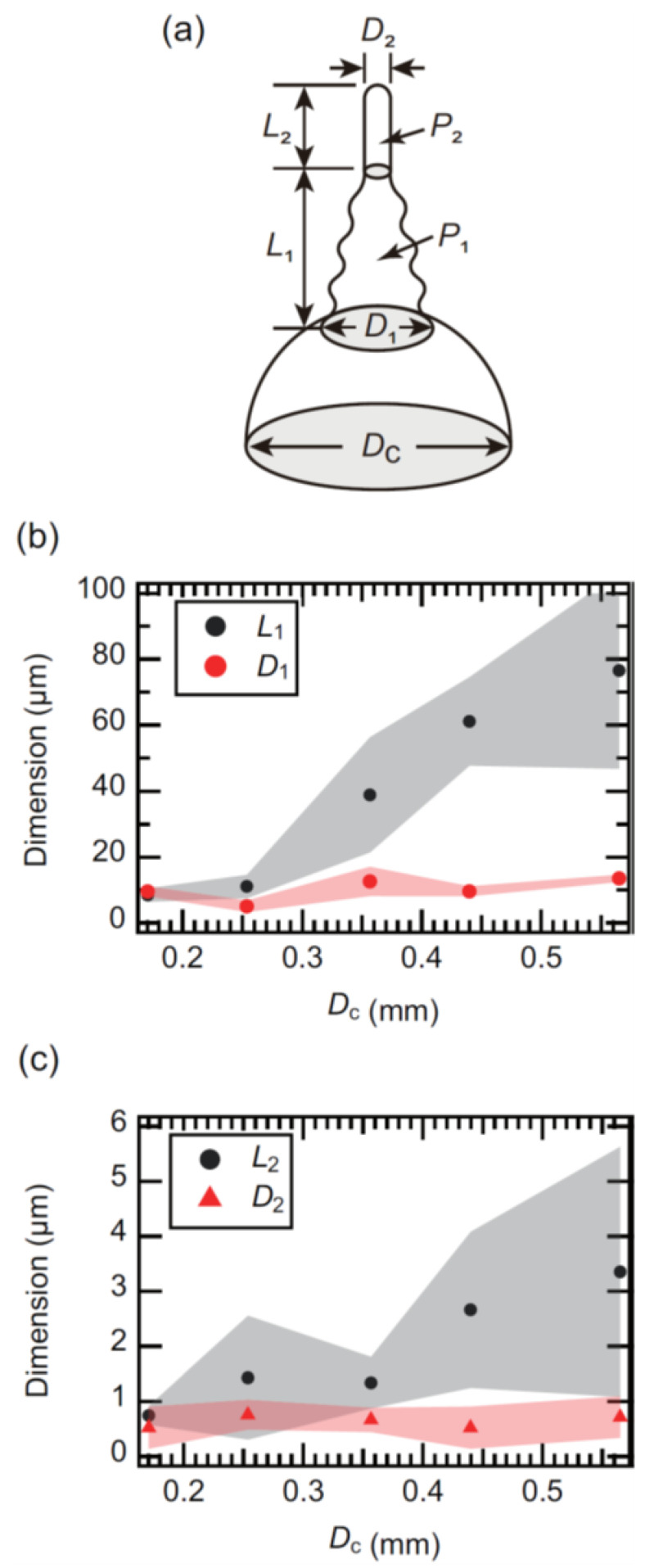
(**a**) Schematic illustration of the typical EgaIn corn and the protrusion with geometric classification. (**b**) The measurement results of the relationship between *L*_1_, *D*_1_, and *D*_c_. (**c**) The measurement results of the relationship between *L*_2_, *D*_2_, and *D*_c_.

We then examined the dimension of the obtained structure as a function of *D*_c_. As mentioned above, there are two regions in the protrusion grown on the EGaIn cone, shown in Figure 3a: the tapered bamboo-like structure and the cylindrical NWs were labeled *P*_1_ and *P*_2_, respectively. The lengths of *P*_1_ and *P*_2_ are denoted as *L*_1_ and *L*_2_. Additionally, the diameter of the disk at the interface of *P*_1_ and the cone was labeled *D*_1_. The diameter of *P*_2_ was determined as *D*_2_. Figure 3b shows *L*_1_ and *D*_1_ as a function of *D*_c_. The hatching areas in Figure 3b indicate the errors. We found that *L*_1_ increases with *D*_c_ and that *D*_1_ was almost independent of *D*_c_. Figure 3c shows *L*_2_ and *D*_2_ as a function of *D*_c_. It exhibits a similar trend, as shown in Figure 3b. The average and minimum diameters of *P*_2_ were approximately 0.6 and 0.2 µm. Therefore, we found that the size of the mask hole could control the length of the protrusion.

In general, in order to minimize the surface energy, the surface area must be minimized. In other words, splitting a droplet should result in the formation of two hemispherical droplets. However, even though the EGaIn used in this study is a liquid, sub-μm diameter wires were observed in the splitting region after the droplet was split. It can be said that the liquid changed to a solid-like phase during the pulling process in order to become an anisotropic quasi-one-dimensional shape. The reason for this is discussed as follows.

**Figure 4:**
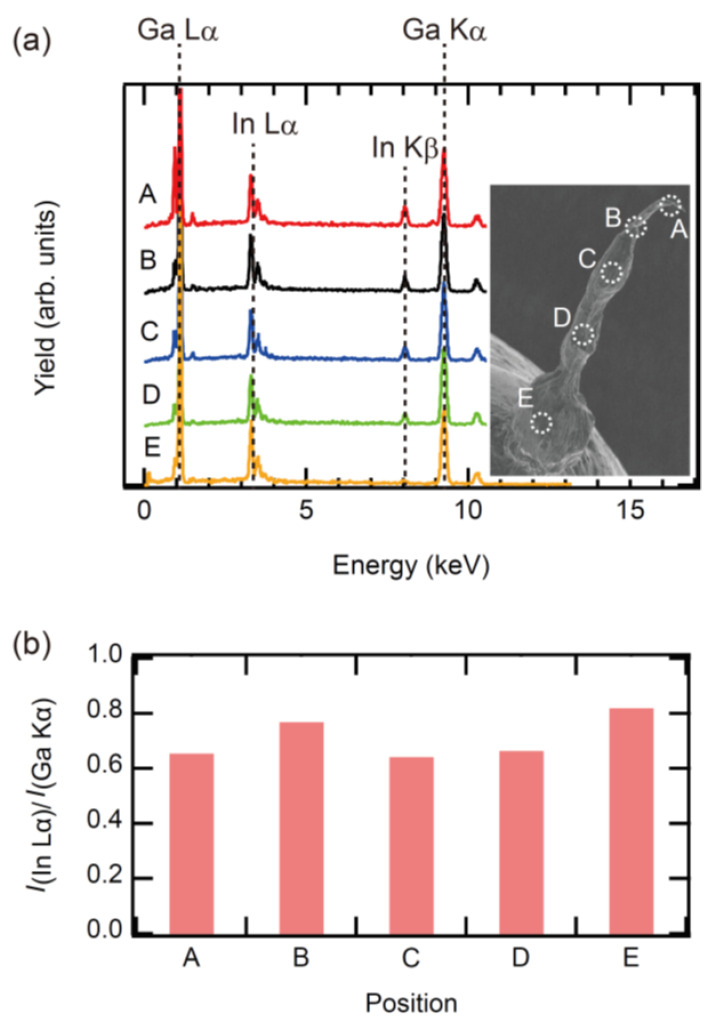
(**a**) Elemental composition from tip part to end part of EGaIn protrusion. (**b**) The intensity ratio of In to Ga from tip part to end part of EGaIn protrusion.

One of the possible reasons why the material phase of EGaIn droplets changed from liquid to solid is that the composition shifted from the eutectic point due to mechanical stress; according to Anderson’s Ga-In binary phase diagram [30], the melting point increases when In increases or decreases from the eutectic point. In other words, a change in the In/Ga ratio from the eutectic composition is thought to have caused the melting point of EGaIn to rise above RT. Therefore, we investigated the elemental composition of the anisotropic structure of the tip using EDX; we measured the ratio of In to Ga at various locations in the *P*_1_ and *P*_2_ regions. Figure 4a shows the EDX spectra from five points of the wire. The inset of Figure 4a shows the points where the measurements were made. In all spectra, the prominent peaks originating from In and Ga were observed. No peaks of other elements were observed; in order to clarify the compositional ratio of In and Ga, we estimated the intensity ratio of InLα to GaKα (Figure 4b). No significant difference was observed between the measurements at any of the locations. Therefore, since the In/Ga ratio did not change from place to place, we can say that solidification due to compositional ratio shift is not the reason for the formation of NWs.

Another possible reason is the presence of native oxide skin on the EGaIn surface. In general, EGaIn in air consists of a liquid core and an atomically thin Ga oxide (β-Ga_2_O_3_) shell [1,2]; when EGaIn comes into contact with oxygen, an oxide film with a thickness of about 3 nm is known to form. In this study, EGaIn was stretched in the air, and it is thought that the surface oxide film was formed simultaneously during the stretching process of the EGaIn droplet by an external force, resulting in the structure of the Ga oxide shell filled with liquid EGaIn. Since the oxidation rate might be faster than the stretching rate, it is assumed that the oxide skin was formed immediately during stretching and breakup. Since gallium oxide is solid and has a tubular structure, the NWs are expected to be mechanically stable even though there is liquid in the anisotropic shape. We could not confirm the presence of the oxide film in this study; therefore, further analysis is needed in the future. As mentioned above, we believe that EGaIn NWs can be formed by a combination of the Plateau–Rayleigh instability and in situ oxidation of the EGaIn during pulling and breakup.

In conclusion, we coined a simple method to fabricate liquid metal NWs using PDMS substrate, cellophane film mask, and an EGaIn droplet. This process is based on the Plateau–Rayleigh instability. When an EGaIn droplet was pulled and broken down, a NW was formed on a “beads-on-thread”-shaped anisotropic microstructure grown on the droplet. It was found that the length of the NW could be controlled by the size of the hole in the mask. In the future, we will expand the method developed in this study to other materials to confirm its versatility. Then, we will try to apply the liquid metal NWs to the three-dimensional wiring of integrated circuits and tips of scanning probe microscopes.

## Data Availability

The data presented in this study are available on request from the corresponding author.
